# Ecolabels and Sustainability in the Seafood Sector: Key Elements of the Debate and Shortcomings

**DOI:** 10.1021/acsenvironau.5c00019

**Published:** 2025-06-04

**Authors:** Sandra Ceballos-Santos, Eva Martínez-Ibáñez, Jara Laso, Alba Bala, Pere Fullana-i-Palmer, María Margallo, Rubén Aldaco

**Affiliations:** † Department of Chemical and Biomolecular Engineering, University of Cantabria, Av. de Los Castros s/n, Santander 39005, Spain; ‡ UNESCO Chair in Life Cycle and Climate Change, Escola Superior de Comerç International (ESCI), 685983 Universitat Pompeu Fabra (UPF), Passeig Pujades 1, 08003 Barcelona, Spain

**Keywords:** Sustainable consumption, certification schemes, consumers, fisheries, aquaculture

## Abstract

The seafood sector plays a key role in global nutrition but is confronted with significant sustainability challenges including overfishing, marine debris, and the impacts of climate change. In response, several measures have been implemented, such as the introduction of fishing quotas, restrictions on fishing zones, expansion of aquaculture, increased monitoring, and promotion of sustainable consumption. In this context, ecolabels are recognized as tools to encourage sustainable consumption by influencing consumer behavior. However, their effectiveness is hindered by limited consumer awareness, regulatory inconsistencies, and incomplete integration of environmental and social impacts into their criteria. In this Perspective, we explore how these key challenges are incorporated into ecolabel standards and evaluate their potential to influence consumer behavior toward sustainable choices Through a review and insights from a life cycle perspective, we identify critical gaps in current ecolabeling schemes, such as a lack of representativeness, incomplete evaluation, and unclear or nonintuitive communication to consumers, and outline a potential roadmap for their improvement. Addressing these gaps is essential for fostering trust and advancing sustainability in the seafood sector.

## Graphical abstract

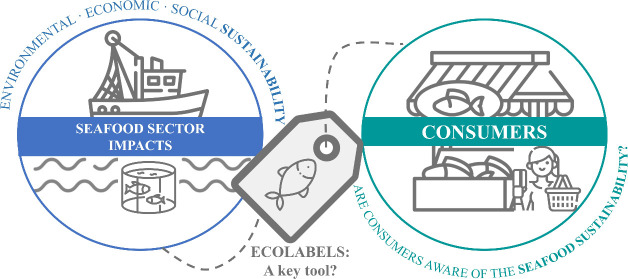

## 1 Introduction

Seafood supply chains are essential to global food security, providing over half of the world’s population with at least 15% of their dietary animal protein as of 2022.^1^ Driven by population growth and demographic shifts, the output from fishing and aquaculture is projected to grow by over 41% in the coming decade.^1^ This increasing demand presents a dual challenge for the global seafood industry: meeting rising demand for nutritious,^2^ sustainable food while mitigating environmental impacts such as overfishing,^3^ habitat degradation, greenhouse gas emissions, and plastic pollution.^4^ The seafood sector also holds immense socio-economic importance, generating 61 million jobs worldwide, supporting the livelihoods of 7% of the global population and contributing substantially for 10% of global agricultural exports, providing 185 million tons of seafood in 2022.^1^

To address sustainability concerns associated with seafood production, certification schemes and environmental labels, namely ecolabels, have been introduced as tools to guide consumer choices^5^ and incentivize sustainable practices among producers.^6^ These schemes, exemplified by the international programs Marine Stewardship Council (MSC) and Friend of the Sea (FOS), aim to raise environmental awareness, ensure compliance with sustainability standards, and promote responsible consumption. However, questions remain regarding their effectiveness, transparency, and ability to genuinely influence consumer behavior. Concerns about greenwashing and the proliferation of misleading ecolabels underscore the need for critical evaluation and improvement of these programs.

This Perspective explores the literature on certification schemes and ecolabels in the seafood industry, aiming to illuminate their role in addressing sustainability challenges and guiding consumers. Specifically, the study examines (1) the environmental and socio-economic challenges in the seafood sector, (Section 2); (2) the communication of these issues through certification schemes (Section 3); and (3) the limitations of ecolabels in driving sustainable consumer behavior (Section 4). Given the global significance of sustainability in the seafood sector and growing consumer interest in ethically sourced products, the findings of this study are intended to guide policymakers, industry stakeholders, and consumers in more sustainable practices and informed decision-making.

## 2 Sustainability Challenges in the Seafood Sector

The term ″seafood sector″ encompasses all activities related to capture and cultivation, processing, distribution, marketing, and consumption of food derived from marine and other aquatic sources.^4^ It typically covers a wide range of products, including freshwater and marine finfish species (such as salmonids, cod, hake, tuna, seabass, and seabream), as well as shellfish (e.g., cephalopods), crustaceans (e.g., shrimps and prawns), and algae (both macro and micro).^7^ Historically, fisheries have been the main sources of seafood production. However, the overexploitation of various marine species, the increasing demand for accessible and sustainable protein sources, and technological advances have led to a gradual shift towards aquaculture.^8^ Nevertheless, aquaculture also has a range of significant environmental impacts.

Sustainability in the seafood sector is an overarching goal that requires a balanced focus on three fundamental pillars: environmental, economic, and social.^9^ These pillars are not only interdependent, but also crucial for ensuring the long-term development and viability of the sector.^5^ The environmental component addresses vital issues such as overfishing, adaptation to climate change, and management of marine debris, which are essential for the conservation of aquatic ecosystems.^10^ The economic aspect focuses on achieving long-term viability by balancing profitability and sustainability to maintain market stability. Finally, the social dimension emphasizes the protection of labor rights and worker welfare, ensuring fair and equitable working conditions as well as ensuring generational succession in the context of a shortage of workers. These main dimensions are summarized in Figure 1 and explored in more detail in the coming sections.

**Figure 1:**
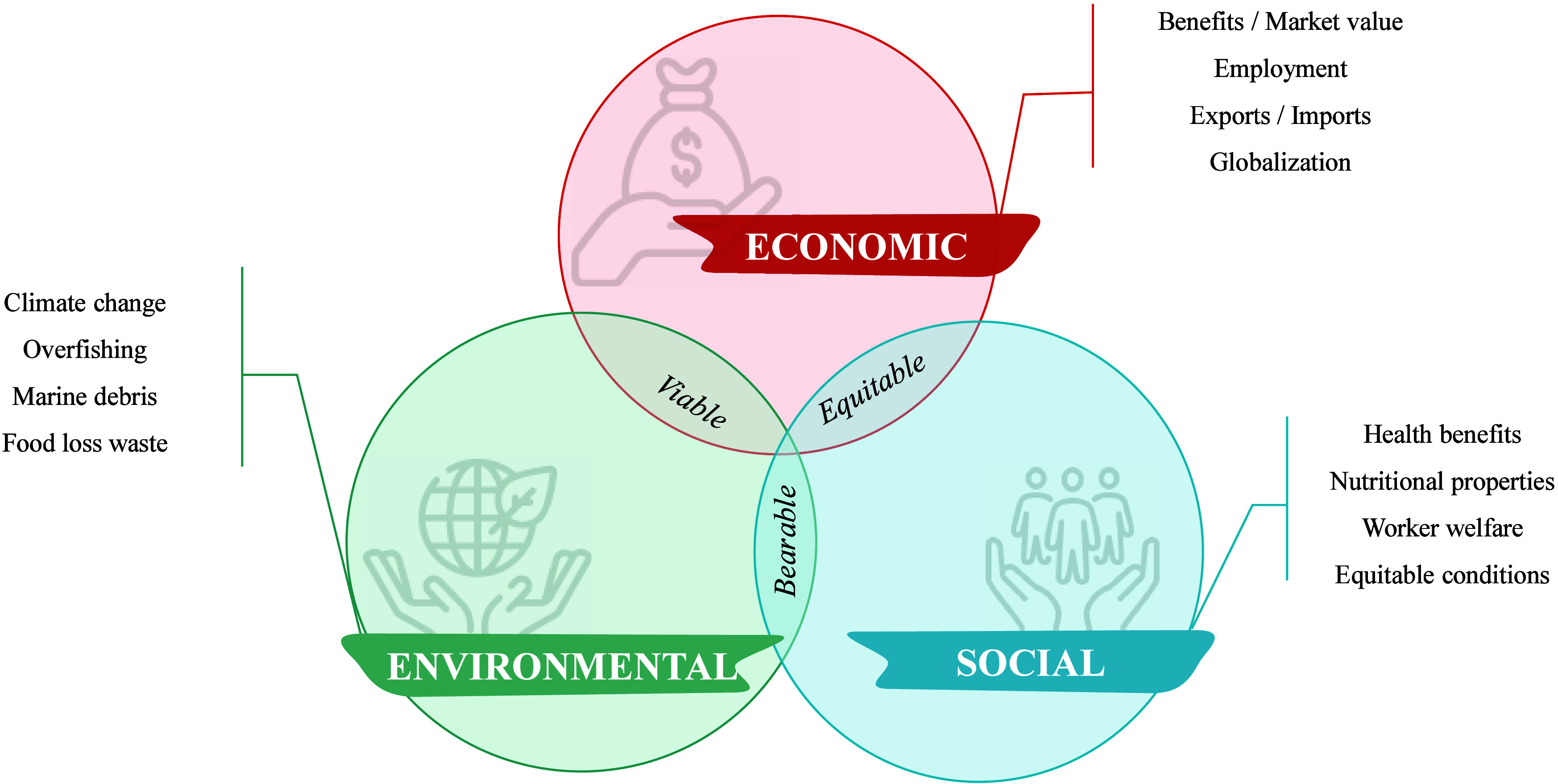
Sustainability dimensions and their relevance in fisheries.

### 2.1 Socio-economic Perspective: The Role of the Seafood Sector in the Global Economy

Social sustainability in the seafood industry refers to the integration of economic, social, nutritional, and cultural factors into management to ensure the well-being of fishing communities and workers in the sector. In 2022, approximately 223 million tons of seafood were produced globally, including both wild fisheries and aquaculture. For the first time in history, aquaculture (51%) exceeded wild capture (49%) in terms of production volume.^1^ In economic terms, the seafood sector generated a total sales value of 472 billion USD in 2022.^1^ China leads the sector, accounting for 36% of global fish production, followed by other Asian countries (34%), America (10%), Europe (10%), Africa (7%), and Oceania (1%). Seafood is also the most traded food commodity globally, with 38% of aquatic animals entering the international market in 2022.^1^

The seafood sector employed 62 million people in primary activities, including full-time, part-time, and occasional workers.^1^ However, this sector, particularly industrial fishing, has been reported for labor abuses, including the exploitation of migrant workers, lack of formal employment contracts, and hazardous working conditions.^11^ Ensuring fair wages and decent labor conditions is essential for achieving social sustainability in the sector, which requires stronger regulation, increased transparency, and the effective enforcement of international labor rights standards.^1^

Seafood is widely recognized by the research community as a highly nutritious animal-source food,^12^ offering high-quality protein and a rich nutritional profile that includes fundamental fatty acids (omega-3 polyunsaturated acids), vitamins (A, B, and D), and minerals necessary for human health.^2^ Reflecting this, the global supply of aquatic animal foods has increased globally at a faster rate than that of annual population growth. Specifically, between 1961 and 2022, global per capita consumption of aquatic animal foods increased at an average annual rate of 2%. In 2022, the average per capita consumption of aquatic animal foods reached 20.7 kg, accounting for approximately 15% of the animal protein supply for the global population.^1^ However, it is important to note that average consumption is strongly influenced by regional factors and other variables such as availability, accessibility, seasonality, and cultural and individual preferences. For instance, some countries, such as Spain, have experienced a negative trend in seafood consumption. Notably, the consumption of fresh seafood and fish in Spain declined by approximately 34.3% in 2023 compared to 2008.^13^ This decline may be attributed to changes in dietary habits, rising prices, cultural factors, and lifestyle choices, among other reasons.

### 2.2 Environmental Dimension: Key Environmental Issues Facing the Seafood Sector

The primary threats to oceans, and consequently to fisheries and the entire supply chain, include pollution (such as marine debris (MD) in water and emissions to air, including greenhouse gases, nitrogen oxides, and particulate matter), overfishing, food loss and waste (FLW), and the impacts of climate change.^14^ Addressing these critical issues is integral to achieving the United Nations Sustainable Development Goals (SDGs), which provide a “call for action by all countries—poor, rich, and middle-income—to advance prosperity while protecting the planet”.^15^ Specifically, SDG 14, “Conserve and sustainably use the oceans, seas, and marine resources”, promotes the conservation and sustainable use of these water bodies, which are responsible for producing half of the oxygen we breathe, absorbing around 30% of the annual CO_2_ emissions generated by human activities, and providing part of the animal protein we consume daily.^16^ Similarly, SDG 12, “Responsible consumption and production”, highlights the importance of sustainable production and consumption patterns, advocating for a reduction in the environmental impacts associated with food production and a decrease in food waste generation.^9^

Focusing on the in-situ effects of fisheries and related activities, the Food and Agriculture Organization (FAO) is particularly concerned about the state of the global seafood sector^1^ and its environmental impacts on marine ecosystems and biodiversity.^17^ The expansion of fishing activities in recent decades has negatively impacted many fish production systems, and combined with weak management, regulation, and illegal fishing,^18^ has led to the overexploitation of many fish populations.^19^ According to the FAO, approximately 37.7% of fish populations are fished at biologically unsustainable levels.^1^ To combat this phenomenon, many countries have implemented policies that restrict fishing levels to stabilize catch rates and ensure resource productivity.^20^ Aquaculture industry is also associated with eutrophication of aquatic ecosystems, introduction of non-native species, alteration of local ecosystems, escape of farmed species, biotic depletion, fish diseases or parasites, and the intensive water use, among others.^21^

Furthermore, carbon dioxide (CO_2_) concentrations, along with other greenhouse gases (GHGs), have increased by 40% since pre-industrial times, primarily because of fossil fuel emissions from human activities.^22^ Climate change drives ocean warming, oxygen depletion, expansion, and acidification, placing increasing stress on aquatic systems that support fisheries and aquaculture and heightening the risk of species extinction.^14^ The ocean has absorbed 93% of this excess heat and sequestered 30% of anthropogenic CO_2_ emissions.^22^ Rising temperatures are reducing dissolved oxygen levels in marine environments, forcing many species to migrate toward higher latitudes and deeper waters in search of more favorable conditions. Simultaneously, the absorption of increased CO_2_ is leading to ocean acidification, with potentially detrimental effects on aquatic ecosystems.^9^ Additionally, rising sea levels pose a threat to vital coastal ecosystems, such as mangroves and coral reefs, which play a crucial role in supporting marine biodiversity.

In addition to the previously mentioned in situ effects, such as the extraction of target and non-target species, overexploitation, changes in marine trophic networks, and other alterations to marine ecosystem structures, it is essential to consider the indirect and off-site impacts of fishing activities.^23^ Indirect environmental impacts are typically associated with the extraction and transformation of natural materials and fossil fuels used in the construction, use, and maintenance of fishing units.^12^ These include emissions from fuel combustion, the release of antifouling substances, the use of refrigerants, the loss of fishing gear, the discharge of wastewater and waste, and the release of cleaning agents, among others, as shown in Figure 2. Specifically, the fishing sector contributes approximately 1.2% of global oil consumption, resulting in an estimated 134 million metric tons of CO_2_ equivalents emitted into the atmosphere.^24^

**Figure 2:**
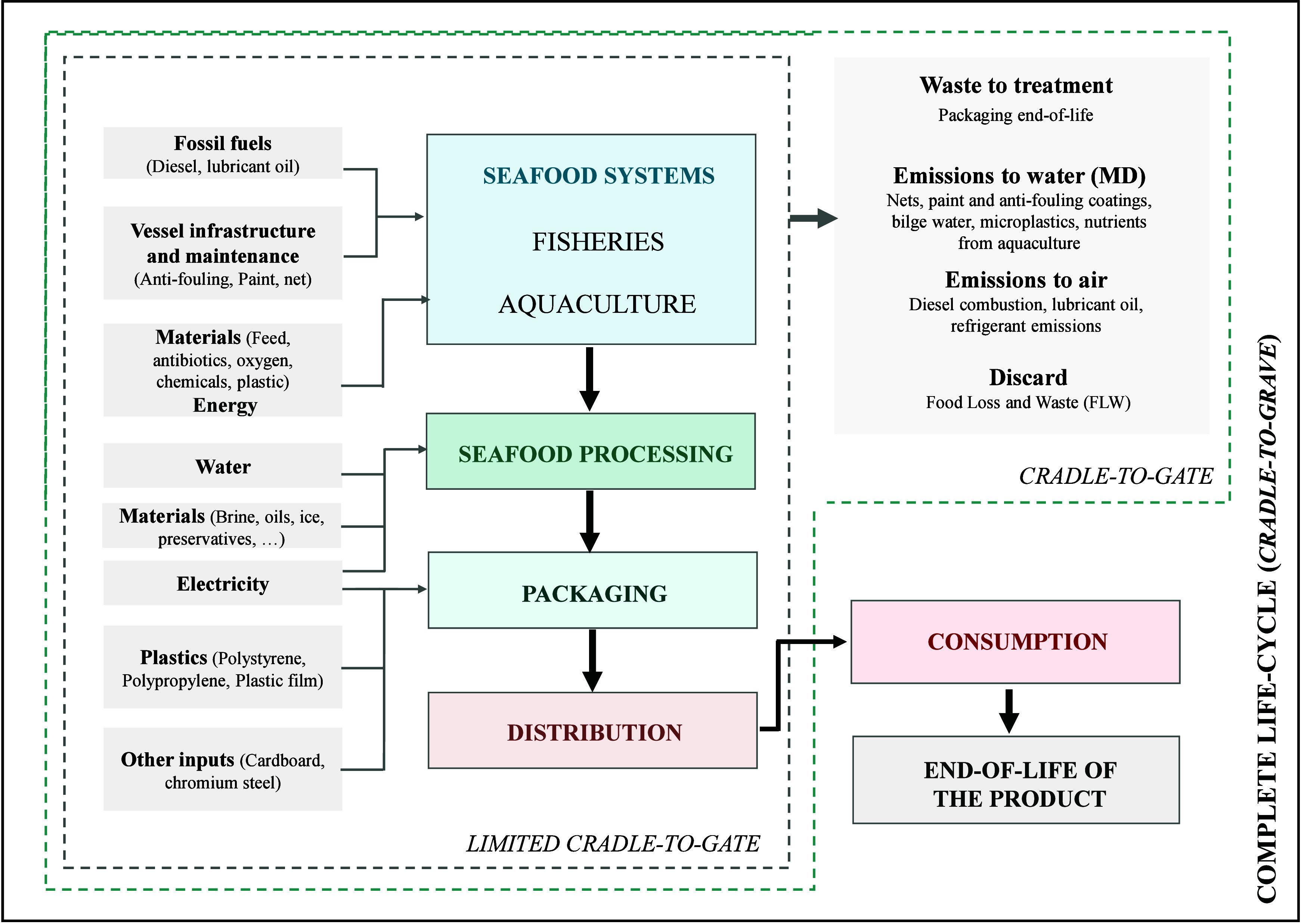
Depiction of resource consumption and environmental impacts in the seafood sector using a “cradle-to-grave”’ flow diagram.

Another significant challenge faced by the fisheries sector in the context of indirect impacts is MD, which refers to any persistent solid material discarded, abandoned, or lost in the marine environment, such as plastic materials (macroplastics, microplastics, or ghost nets), metal materials (ship parts, cans, or discarded metal structures), or hazardous waste (batteries, chemicals, oils, or fuels). In particular, pollution from plastic waste (both microplastics and macroplastics) presents a critical challenge for the fisheries sector.^25^ A study has estimated that between 4.8 and 12.7 million tons of plastic waste entered the oceans in 2010,^26^ adversely affecting marine biodiversity and consequently human health.^27^ A substantial proportion of marine debris originates from fishing activities, including the loss of fishing gear and poorly managed plastics at the end of their life cycle, both of which are classified as macroplastics.^28^ Additionally, examples of microplastics released during fishing activities include marine coatings applied to boats, which can leach into the environment during fishing operations; plastic remnants that can be lost during the production of plastics; and particles from tire abrasion during these activities.^25^ The presence of microplastics in the ocean not only affects marine organisms, but also poses risks to human health for those who consume seafood.^29^ To ensure sustainability of fishing activities, it is essential to minimize plastic use and control losses occurring throughout the supply chain. In this regard, mapping plastic flows across the product’s life cycle is crucial. Additionally, the use of biodegradable materials in fishing gear manufacturing has been proposed as a potential solution to mitigate the environmental impact of lost or discarded fishing equipment.^30^ These materials degrade more rapidly in the marine environment through processes such as hydrolysis and microbial biodegradation,^31^ thereby reducing plastic pollution. However, their higher cost and lower durability compared with conventional plastics present challenges to widespread adoption, highlighting the need for regulatory measures and economic incentives.

Finally, FLW has emerged as a major social and political issue, with over one-third of global food production wasted along the food supply chain.^32^ In the fisheries sector, this loss is particularly acute, accounting for approximately 35% of global production each year.^33^ The FAO defines “food loss” as the decrease in the quantity or quality of food resulting from decisions and actions taken by the food industry.^34^ In contrast, “food waste” refers to all edible and inedible fractions of seafood raw materials that are discarded throughout the food supply chain, stemming from decisions and actions made by primary producers, retailers, food service providers, and consumers. Several factors contribute to FLW within the seafood sector, including capture methods that lead to the unintended capture of non-target species that are subsequently discarded, inefficiencies in the cold chain, inadequate or oversized packaging, rejection of products that do not meet quality standards, and surplus unsold products, among others.^35^

The waste of natural resources exerts increasing pressure on marine ecosystems and contributes to the depletion of fish stocks. In a context where climate change is already reducing species availability, these losses are even more critical.^7^ Therefore, there is an urgent need for sustainable practices throughout the seafood supply chain that incorporate innovative strategies to valorize seafood waste into value-added products.^3^ These products can include biochemicals, biomaterials, and biofuels, thereby generating value-added products that provide economic benefits while minimizing environmental impacts.^36^

To better understand environmental impacts and ensure sustainability of the seafood industry, it is essential to develop an integrated, science-based approach to impact assessment. In this context, Life Cycle Assessment (LCA) has emerged as a widely accepted and robust tool for quantifying the environmental impacts of seafood production throughout its entire life cycle. LCA provides a comprehensive, quantitative, and objective framework for analysis.^37^ This methodology enables the identification of opportunities to improve environmental performance and informs decision-makers about the environmental impact of products, product systems, and their alternatives.^38^ As shown in Figure 2, this approach allows the analysis of the environmental burdens associated with each stage of the seafood sector’s life cycle, from raw material extraction to end-of-life. Depending on the stages considered, the study may adopt a ″cradle-to-gate″ approach or a ″cradle-to-grave″ approach if the consumption and end-of-life stages are included.

Additionally, LCA supports the selection of sustainability indicators and can be applied for marketing purposes, enhancing the legitimacy and transparency of eco-labels for consumers and regulators. By doing so, it helps mitigate the risks of ″greenwashing″.^39^ Over the past few decades, numerous studies have explored the application of LCA in the fisheries sector, with notable contributions, including seminal reviews by Vázquez-Rowe and colleagues^39^ and a more recent one by Ruiz-Salmón et al.^10^ These studies highlight the importance of LCA as a tool for guiding sustainable practices and decision-making in fisheries and aquaculture, ensuring that environmental considerations are integrated into production strategies and contributing to more sustainable food systems.

## 3 Certification Schemes and Ecolabels: Bridging the Gap in Seafood Sector Sustainability Awareness

As previously highlighted, the seafood sector faces significant challenges across the three pillars of sustainability—environmental, social, and economic—that are critical to its long-term viability. In response, there is a growing emphasis on effectively communicating the sustainability attributes of seafood products to consumers. This trend has heightened pressure on producers to adopt verifiable, transparent, and sustainable practices that align with global sustainability goals.^5^ Certification schemes and ecolabels provide valuable opportunities to enhance transparency, traceability, and sustainability in fishing and aquaculture operations. However, their effectiveness is often hindered by challenges such as the proliferation of uncontrolled and misleading ecolabels, a common issue in supermarkets in industrialized countries.^40^ Addressing these shortcomings requires fostering close collaboration among stakeholders to ensure that consumers have access to accurate, science-based information. Persistent questions remain about whether current certification schemes effectively meet the needs of both producers and consumers and how these labels can be improved to promote sustainable consumer behavior. This section aims to address these issues by critically reviewing ecolabels from a scientific perspective, providing insights and recommendations to strengthen their impact.

### 3.1 State of the Art

A bibliographic search was conducted to touch base with the current literature on ecolabels and sustainability certifications in the fisheries and aquaculture sectors. The search was conducted in the Scopus database,^41^ using key terms such as ’fish*’, ’aquaculture’, or ’seafood’, combined with ’ecolabel*’, ’environmental certification’, and ’consumer’. Articles were required to contain these terms in the title, abstract, or keywords. Given the anticipated high volume of results, the search was refined to focus on publications from the past ten years, specifically covering the period from 2014 to 2024. This time frame was chosen to capture recent trends in the subject matter while excluding outdated research. Only peer-reviewed literature, including articles, reviews, and books published in English, was considered. In addition, the search was limited to the subject area of environmental science, focusing the results on materials addressing the topic from an environmental perspective. In total, 62 contributions were identified, comprising 4 review articles, 2 book chapters, and 56 research articles. A multi-step screening process was applied to categorize and evaluate the articles based on their relevance to the study’s objectives (Figure 3). Finally, a sample of 32 papers was selected to examine key contributions to the research question.

**Figure 3:**
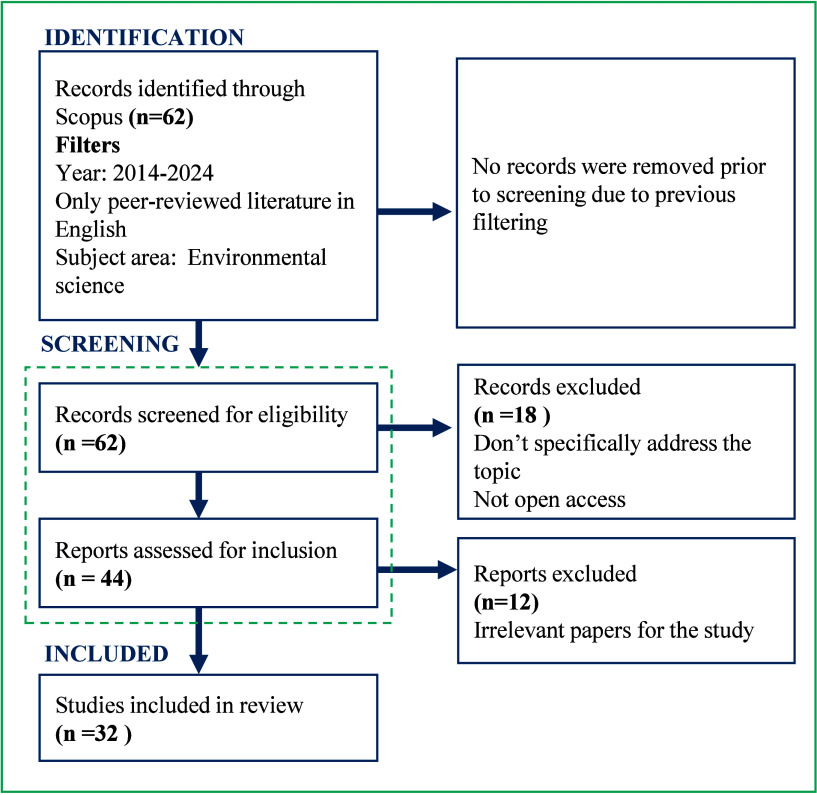
Literature search strategy.

Table 1 summarizes the reviewed articles, providing a concise overview of the research they encompass.

**Table 1:** Compilation of Articles Consulted Categorized by Region, Ecolabels, And Main Topics Discussed^a^

		Ecolabels					Key focus and insights				
Reference	Region	FOS	MSC	ASC	Dolphin Safe	Others	Consumers perception	Producers’ perception	Price premiums (WTP)	Framework weaknesses	Regulations
[^42^]	Global	X	X	X			X			X	X
[^43^]	Global		X				X			X	X
[^5^]	Global		X			X	X		X		X
[^40^]	Global	X	X		X		X			X	X
[^44^]	United States		X				X		X		
[^45^]	Germany			X		X	X		X		
[^46^]	Germany		X	X			X		X		
[^47^]	Switzerland		X	X		X	X			X	
[^48^]	Germany		X	X			X		X		
[^49^]	Germany		X				X		X		
[^50^]	Denmark, Italy, Ireland, Germany, Greece, Norway, Poland, Spain and the United Kingdom			X		X		X		X	X
[^51^]	Italy	X			X		X		X		
[^52^]	United States		X	X			X		X		
[^53^]	Italy					X	X				
[^54^]	Global	X		X		X	X				
[^55^]	Norway and United Kingdom		X	X			X	X	X		X
[^56^]	United States and United Kingdom	X	X		X		X			X	
[^57^]	Sweden		X	X		X	X				
[^58^]	Korea		X	X			X				
[^59^]	United States		X		X	X	X		X	X	X
[^60^]	Europe		X	X	X		X	X			
[^61^]	Italy					X	X		X		
[^6^]	Greece, Italy and Spain	X	X		X	X	X				
[^62^]	Europe	X	X	X		X	X				
[^63^]	Australia	X	X			X	X	X		X	X
[^64^]	Asia					X				X	X
[^65^]	Australia		X	X		X	X			X	
[^66^]	Japan	X	X	X				X		X	X
[^67^]	Taiwan		X		X		X	X		X	X
[^68^]	Canada	X	X			X	X	X		X	X
[^69^]	United States	X			X	X		X		X	X
[^70^]	Canada	X	X	X			X		X	X	

a The column *“Ecolabels”* lists the certifications mentioned in each study. The column *“Key focus and insights”* categorizes the research into five main topics: (1) *Consumer perception* refers to studies based on surveys or research exploring consumer opinions regarding ecolabels; (2) *Producer perception* includes studies addressing the perspectives of producers of fish or aquaculture products; (3) *Price premiums and willingness to pay* covers research examining the price attributes associated with ecolabels; (4) *Framework weaknesses* highlights studies evaluating ecolabel schemes and identifying existing gaps or shortcomings; and (5) *Regulations* refers to discussions on the standardization and regulation of certification schemes, including aspects of government oversight and incentives.

#### 3.1.1 Review Articles: Key Elements of Focus

An extensive analysis of the published reviews was conducted as the initial step to ascertain the distinct scopes and overarching conclusions drawn from each study. The first review within the evaluated period conducted by Micheli et al. proposed a conceptual system-wide fisheries and aquaculture certification program aimed at promoting more sustainable and resilient seafood production involving various stakeholders.^42^ The authors highlighted the ineffectiveness of existing certification programs, which often overlooked entire marine ecosystems and human societies reliant on them. Key barriers included high financial costs, extensive data requirements, and fixed thresholds that hindered the participation of small-scale fisheries and producers. These challenges were similarly highlighted by Wakamatsu and Wakamatsu^66^ and further explored from a producer perspective in a study by Chikudza and colaborators.^50^ By examining producers’ perceptions of the costs and benefits of ecolabelling and investigating the influence of operation scale on these perceptions, they found that producers viewed ecolabelling as offering significant opportunities. These included enhanced access to local and export markets, improved product acceptance, potential price premiums, long-term supply contracts, greater investment attractiveness, positive consumer perception of aquaculture products, and an increased industry reputation. However, producers also reported notable challenges, particularly high compliance costs, expensive and time-consuming annual audits, and uncertainty regarding long-term financial benefits.

Despite these constraints, studies have demonstrated the ability of ecolabels to increase the marginal willingness-to-pay (WTP) for products sourced from developing countries and small-scale systems.^59^

Barclay and Miller offered a different perspective, suggesting that the sustainable seafood movement should be viewed as a ″governance concert″ involving various stakeholders, including consumers, rather than relying solely on consumer-driven approaches like WTP studies.^43^ Maesano et al.,^5^ Ankamah-Yeboah et al.,^45^ and Fonner and Sylvia^52^ focused on consumers’ WTP for sustainability-labeled fish products and identified ″origin″ and “local labels” as the most influential factor in consumer choices, commanding the highest price premium, but also noted the challenges of consumers recognizing and interpreting ecolabels.

Finally, Giacomarra and colleagues reviewed literature on two major private and voluntary ecolabels in the fish industry—FOS and MSC.^40^ Their analysis proposed a framework to promote sustainability by engaging certified fisheries, retailers, and public authorities, addressing key governance and organizational challenges. The authors emphasized the importance of fish companies providing detailed information on the impact of ecolabels on marine ecosystems to influence consumers’ purchasing decisions effectively. Furthermore, they argued that marketing efforts should go beyond merely publishing sustainability information on corporate Web sites, as is commonly done. Instead, firms should implement educational initiatives at points of sale—such as supermarkets and fish markets—where consumers make their purchasing decisions, ensuring greater awareness and engagement.

The diversity of key topics identified in the overview evidenced the complexity of the issue. While progress has been made over the past decade and research on specific topics has contributed to the understanding of sustainability in fisheries and aquaculture, significant gaps remain that impede meaningful progress toward more sustainable practices. Although there is a clear need for certification standards, these often disadvantage small-scale fisheries and producers. This underscores the need to re-evaluate the criteria, thresholds, and costs associated with certification. Targeted incentives for small producers could include subsidized certification fees, training, fishery improvement projects access to premium markets, low-interest loans, tax benefits, and streamlined certification processes.^71^ Partnerships with cooperatives, non-governmental organizations (NGOs), and local governments, as well as public recognition, would further help reduce costs, increase market access, and support sustainable practices.

While consumers demonstrate a commitment to sustainability and a WTP for sustainable products, it is essential that ecolabels are recognizable and easy to understand and provide verifiable information that confirms the sustainability of these products.

#### 3.1.2 A Focus on Ecolabels through the Consumer Lens: Worldwide Perceptions and Insights

A wide body of literature examines consumer perceptions of ecolabels, covering case studies from various geographical regions and certification schemes (Table 1). This research reveals issues such as information asymmetry and, in some cases, a lack of consumer understanding, commonly termed ″food illiteracy”. Figure 4 illustrates the key topics of discussion related to seafood ecolabels that will be explored below.

**Figure 4:**
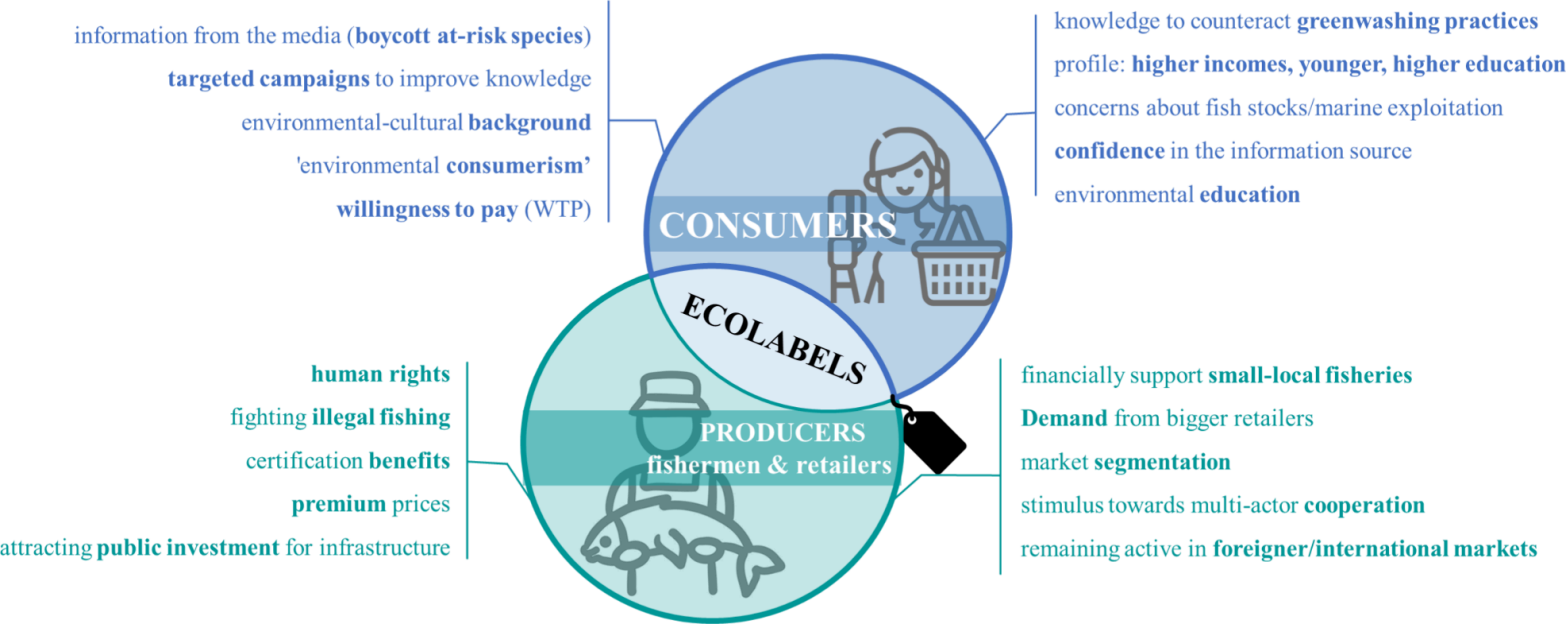
Key elements of discussion surrounding the seafood sector and ecolabels.

Gutierrez and Thornton conducted surveys in the United States and the United Kingdom, revealing that while consumers were somewhat familiar with ecolabels—particularly dolphin-safe and organic—they often struggled to interpret broader ecolabels like MSC or FOS, leading to confusion.^56^ Similarly, Jonell and collaborators demonstrated that in the Sweden context, the recognition and understanding of seafood ecolabels, along with concerns about the negative environmental impacts of seafood production, were the strongest predictors of consumers’ stated intent to purchase ecolabeled seafood.^57^ In contrast, the presence of ecolabels in markets remains relatively low in certain regions, such as western^63^ and south-eastern Australia,^65^ even though some locally caught fish products being certified. In other regions such as Taiwan, research showed that the industry lacks strong motivation to pursue MSC certification, particularly due to cost-sharing implications and limited consumer willingness to pay extra for ecolabel products.^67^ Natali and colleagues explored the potential of ecolabels to enhance the appeal of typically discarded species in Italy.^61^ Their analysis found that consumer interest in these species increased when specific information about ecolabels and the practice of discarding was provided.

Regarding consumer preferences for wild versus farmed fish, Bronnmann and Asche found that the introduction of the ASC label in Germany could reduce the preference for wild fish.^48^ The ASC label addresses negative perceptions of aquaculture, suggesting that sustainability concerns often outweigh quality considerations in shaping consumer choices. Furthermore, Asche et al. found that the ASC is associated with a statistically significant price premium.^46^ In contrast, Forleo and Palmieri observed that in Italy, ecolabels and other environmental attributes had relatively little impact on farmed canned tuna purchases,^53^ suggesting limited influence of sustainability labeling in this market. In the Norway and UK markets, ASC-certified salmon faced criticism for not meeting sufficiently high standards to be considered truly ″sustainable″.^55^ In fact, the certification was limited to business-to-business transactions and did not reach retail supermarket shelves.

Regarding the “age” factor, Del Giudice et al.^51^ and Winson et al.^70^ found that younger consumers in Italy and Canada, respectively, are more likely to choose ecolabeled products. This heightened awareness is attributed to the incorporation of environmental education into schools and widespread digital access. In this regard, Teixeira and Silva suggested that school feeding programs could serve as an effective platform to promote healthy and sustainable eating habits among young people.^72^

An increasing number of consumers view the inclusion of ethical, social, and environmental (ESE) information on product labels as a requirement rather than a choice, as highlighted by Peiró-Signes and collaborators using 2021 Eurobarometer data.^62^ Aiming to define the buyer profile, a study conducted in South Korea by Kim and Lee revealed that consumers with low price sensitivity were found to be more likely to prefer ecolabeled seafood,^58^ whereas those who prioritized price over environmental attributes tended to have lower preferences for these products. Additionally, they found that consumers who value the origin of seafood were more inclined to choose ecolabeled options.

One of the dual aspects of ecolabels is the “warm glow” effect, the positive emotional satisfaction or sense of moral reward that they can generate. Bronnmann et al. examined this phenomenon in the German market and discovered that while the primary motivation for choosing an ecolabeled product was based on environmentally friendly production,^49^ a significant driver was also the personal satisfaction consumers experience from making environmentally eco-conscious choices. This effect contrasts with more altruistic motivation, where individuals prioritize social and environmental issues and choose to support environmental and social initiatives without expecting any personal gain.^6^ The “warm glow” effect raises a potential concern within the ecolabel market, as it suggests consumers may derive emotional gratification from these labels, even when their actual contribution to sustainability is minimal.

A key topic of discussion is the type and quality of information provided on ecolabels, which, in many cases, are wrongly used as an end rather than a means. Many ecolabels act as ″proxy labels”, using simple language or symbols to indicate that a product is sustainable, effectively replacing the need for consumers to assess sustainability independently with each purchase. However, some researchers argue that this approach is limiting, as it does not empower consumers to make informed, sustainable choices based on their own criteria.^44^ To enhance ecolabel effectiveness, it is suggested moving toward more detailed, informative labels that offer consumers the knowledge needed to evaluate a product’s sustainability themselves. In this regard, critical environmental challenges facing the seafood sector today, such as FLW and marine debris, should be addressed within the certification programs. Consumers should be able to have access to sustainable profile examination of their purchases.

Lucas and colleagues evidenced sustainability claims should be supported across all markets and encompass all attributes (such as packaging, product composition, organic certification, and animal welfare), even if some claims are intended for niche markets.^60^ For instance, Gray et al. noted the limited use of ecolabels in the shellfish industry and emphasized the importance of communicating the ecosystem services provided by shellfish production to consumers to promote it as a highly sustainable source of animal protein.^54^

#### 3.1.3 Regulatory Frameworks and Standards for Ecolabeling

The establishment of frameworks, such as Product Environmental Footprint Category Rules (PEFCRs) for seafood production encompassing both fisheries and aquaculture, as well as seafood products, is crucial for advancing sustainability in the sector. The development of a PEFCR for marine fish started in 2014 as part of the second wave of PEFCR pilot projects. By October 2019, the project had been accepted by the Commission after undergoing public consultation and rigorous review by independent LCA experts. This effort culminated in a draft report providing recommendations for a PEFCR for Marine Fish Products.^73^ While this milestone establishes a pathway for environmental assessment within the seafood sector, it is important to note that broader sustainability aspects—beyond environmental metrics—must also be addressed.

In addition to the PEFCR efforts, other frameworks and proposals are in development. For instance, the PCR for fish and fish products v1.0 ^74^ provides guidelines for assessing different types of fish preparations, such as live, fresh, chilled, frozen or dried; with different considerations for fillets and meat, etc. However, this framework does not cover the entire spectrum of seafood products, such as mollusks and crustaceans, leaving significant gaps in the sector. Similarly, the ISO 22948 guidelines outline the methodology for calculating the CF of seafood specifically focusing on finfish.^75^ While valuable, this guide is limited to CF metrics, overlooking other critical challenges.

Once the rules for determining the environmental performance of seafood products are clearly defined, the next challenge will be translating this technical information into accessible formats for consumers. Communicating these findings effectively—through ecolabels, Environmental Product Declarations (EPDs), or other mechanisms—while simultaneously educating stakeholders on what ecolabels mean, the standards behind eco-certification, and the variability between labels is essential to reduce confusion.^68^ Achieving this requires holistic, accessible, and inclusive definition of ″sustainable fish″ to guide stakeholders and citizens.^69^

Governments and intergovernmental organizations could play a key role by adopting the voluntary governance norms developed by the FAO as a mandatory baseline for sustainable seafood ecolabels.^47^ This approach would ensure that any ecolabel claiming sustainability within national jurisdictions adheres to minimal, meaningful, and verifiable standards.^70^ In this regard, Samerwong and collaborators examined three metagovernance arrangements designed to provide harmonized quality assurance across multiple eco-certification standards for aquaculture in Southeast Asia.^64^ Their findings indicate that these arrangements vary significantly in goals and approaches and do not appear to directly reduce consumer confusion. More importantly, these systems introduce a new competitive space where market, state, and civil society actors vie for influence over regulatory mechanisms, each aiming to steer aquaculture toward more sustainable practices. This example underscores the complexity and challenges of the pathway for establishing standardized regulations in the sector.

On the other hand, EU Regulation 1379/2013 aims to establish standards for labeling and transparency within the EU fishery and aquaculture markets to promote sustainability, market stability, and informed consumer choices. It mandates clear labeling of product origin, catch methods, and production details; supports producer organizations in managing supply sustainably; and enforces consistent quality standards across the European Union.^76^ The regulation also emphasizes data collection for market insights and includes measures to support small-scale fisheries. Nevertheless, future amendments should consider the inclusion of more exhaustive environmental, ethical, and social information to create greater consumer awareness of the consequences of their choices on the marine ecosystem and communities, as confusion persists, as demonstrated by this research.

## 4 Discussion and Conclusions

This Perspective presents a comprehensive perspective on the communication of sustainability in seafood products to consumers. The seafood sector’s sustainability hinges on addressing its most pressing environmental challenges—overfishing, marine debris, and climate change—through effective consumer-facing tools like ecolabels. Certification criteria should be expanded to include explicit metrics for addressing marine plastic pollution, reduction of carbon footprint, and climate adaptation strategies. While many certification schemes currently emphasize sustainable fishing practices and resource conservation, the environmental footprint of seafood production goes far beyond these concerns. For example, plastic pollution from the seafood industry, whether from packaging or from discarded fishing gear, is a growing issue that needs greater emphasis. Including these aspects in certification criteria would not only provide consumers with clearer, more actionable information but also align purchasing decisions with broader sustainability objectives. Such integration would enhance consumer trust and align purchasing behavior with sustainability goals

After reviewing the state of the art regarding challenges in the seafood sector today, it becomes evident that the three pillars of sustainability face significant hurdles in securing their future viability. Socio-economic challenges include the generational turnover crisis in fishing activities and the pervasive issue of illegal fishing, which has far-reaching consequences. Environmental challenges are equally pressing. The sector grapples with the dual role of being both a contributor to and a victim of climate change, coupled with pollution in aquatic and terrestrial environments. Moreover, food loss and waste, exacerbated by global population growth, present serious concerns.

The ultimate goal of the seafood sector is to provide safe, high-quality food to the population. In this context, eco-labels emerge as a critical communication tool, offering assurances of food safety and quality. However, the absence of a cohesive regulatory framework—whether regional, national, or global—has been identified as a significant barrier to coordinated progress. While there are ongoing initiatives and an abundance of guides and recommendations, the sheer diversity and volume of information often confuse consumers. Particularly notable is the lack of robust and detailed information presented through eco-labels.

Among the many eco-labels in circulation worldwide, this study highlights MSC standards for fisheries, ASC standards for aquaculture, and the Friend of the Sea (FOS) label as the most prominent. These labels serve as proxies, signaling that the products have undergone external verification. However, they fall short in offering easily accessible and detailed insights into the certification process. Consumers seeking such information often must conduct independent research through the certifying organizations’ Web sites, making transparency less user-friendly.

The literature review reveals that the scientific community is actively engaged in studying eco-labels. Over the past decade, numerous studies have explored consumer perceptions from various angles. However, a recurring limitation is the lack of representativeness. Most studies focus narrowly on specific marine species, geographic areas, and certification schemes. While they employ a range of statistical techniques and sophisticated models providing quantitative assessment of ecolabel effectiveness, their conclusions—such as whether consumers are willing to pay a premium for eco-labeled products—are often too general to establish broader trends. These studies, therefore, function more as case studies, contributing incremental knowledge rather than offering definitive insights.

Additionally, many well-known eco-labels tend to concentrate on the extraction or production stages of fishing or aquaculture activities, often overlooking the subsequent stages of the product’s life cycle up to the point of consumption. This underscores the importance of incorporating life-cycle analysis methodologies—encompassing both social and environmental dimensions—to comprehensively assess all impacts and ensure that these are accurately reflected in eco-labels. In this line, blockchain technology should be explored as a powerful tool for enhancing transparency, automating certification processes, and ensuring compliance with sustainability standards throughout every stage of the food supply chain. The primary challenge lies in communicating complex scientific information to non-specialist consumers in an accessible manner. The interpretation of eco-labels must be straightforward and intuitive, with transparency and traceability being essential. In this vein, emerging research is focusing on developing ″Nexus labels″ that combine multiple impact metrics, such as carbon, water, and energy footprints, while also integrating the product’s nutritional value.^77^ These innovative approaches aim to bridge the gap between scientific rigor and consumer-friendly communication, representing a promising direction for the future of eco-labels.^78^ Nexus labels, which should ideally cover the main pillars of sustainability—environmental, social, and economic impacts—could play a crucial role in fostering a more sustainable and ethically-conscious marketplace by integrating and standardizing ecolabels with other certifications, such as Fair Trade or Carbon Neutral. These additional labels, which focus on issues such as greenhouse gas emissions and ethical production practices, would work synergistically to provide consumers with a clearer, more cohesive message. This integration ensures that consumers are not overwhelmed with multiple overlapping or complementary labels while also reducing the cost and complexity for producers in obtaining numerous certifications—a barrier that can limit accessibility for smaller producers.

In conclusion, after reviewing multiple studies conducted globally, it remains challenging to draw general conclusions about consumer awareness and understanding of ecolabels. Most papers focus on specific regions, particular species, or individual ecolabels, making it difficult to identify overarching trends across the sector. The findings of the present research are primarily limited to offering a general overview of the current state of knowledge and advancements within the scientific community. The MSC and ASC ecolabels are among the most widely recognized worldwide, with broad consumer awareness. However, despite their popularity, these labels are not without controversy, as they face ongoing debates regarding their standards and effectiveness.

Among the limitations of this study, the diverse focus of the reviewed literature—including consumers, producers, regulations, and species-specific studies—along with the limited scope of some research, often restricted to specific geographic regions or markets—posed challenges in drawing unified conclusions. Additionally, variability in research methodologies and the lack of standardized criteria for evaluating the impact of ecolabels further complicated the synthesis of findings. These limitations underscore the need for future research on the broader impact of ecolabels on consumer behavior, the refinement of certification standards, and deeper exploration of governance mechanisms to address emerging environmental concerns in the seafood sector.
